# Estimating visual field loss from monoscopic optic disc photography using deep learning model

**DOI:** 10.1038/s41598-020-78144-1

**Published:** 2020-12-03

**Authors:** Jinho Lee, Yong Woo Kim, Ahnul Ha, Young Kook Kim, Ki Ho Park, Hyuk Jin Choi, Jin Wook Jeoung

**Affiliations:** 1 Department of Ophthalmology, Seoul National University College of Medicine, Seoul, Korea; 2 Department of Ophthalmology, Hallym University Chuncheon Sacred Heart Hospital, Chuncheon, Korea; 3 Department of Ophthalmology, Seoul National University Hospital, Seoul, Korea; 4 Department of Ophthalmology, Jeju National University Hospital, Jeju-si, Korea; 5 Seoul National University Hospital Healthcare System Gangnam Center, Seoul, Korea

**Keywords:** Glaucoma, Eye diseases

## Abstract

Visual field assessment is recognized as the important criterion of glaucomatous damage judgement; however, it can show large test–retest variability. We developed a deep learning (DL) algorithm that quantitatively predicts mean deviation (MD) of standard automated perimetry (SAP) from monoscopic optic disc photographs (ODPs). A total of 1200 image pairs (ODPs and SAP results) for 563 eyes of 327 participants were enrolled. A DL model was built by combining a pre-trained DL network and subsequently trained fully connected layers. The correlation coefficient and mean absolute error (MAE) between the predicted and measured MDs were calculated. The area under the receiver operating characteristic curve (AUC) was calculated to evaluate the detection ability for glaucomatous visual field (VF) loss. The data were split into training/validation (1000 images) and testing (200 images) sets to evaluate the performance of the algorithm. The predicted MD showed a strong correlation and good agreement with the actual MD (correlation coefficient = 0.755; R^2^ = 57.0%; MAE = 1.94 dB). The model also accurately predicted the presence of glaucomatous VF loss (AUC 0.953). The DL algorithm showed great feasibility for prediction of MD and detection of glaucomatous functional loss from ODPs.

## Introduction

Glaucoma is a chronic optic neuropathy that is characterized by progressive axonal loss and retinal ganglion cell damage^1^. Irreversible visual impairment can occur in cases where appropriate diagnosis and treatment are delayed^2,3^.

Diagnosis of glaucoma is made on the basis of structural information from the optic nerve head and retinal nerve fiber layer (RNFL) and visual field (VF) assessment by standard automated perimetry (SAP). VF assessment is recognized as the important criterion of functional glaucomatous damage judgment; however, it requires patients’ concentration during the test time, and can show large test–retest variability^4,5^. Furthermore, various conditions such as neural noise and deterioration of cognitive function can incur VF test variability even with good reliability indices^6^. Contrastingly, monoscopic optic disc photographs (ODPs) or red-free RNFL photographs taken by experienced technicians are relatively more robust test criteria, though they also can be affected by conditions including media opacity or poor pupil dilation. In this light, prediction of VF test results by structural information certainly would be useful.

A close relationship between optic nerve head morphology and VF status has been reported in previous studies^7,8^. Also, an optic disc staging system moderately correlatable to global indices such as mean deviation (MD) has been proposed^9^. The introduction of various ocular instruments that provide quantitative information on the optic nerve head or RNFL, SD-OCT for example, have enabled more accurate prediction of VF status^10,11^. Nonetheless, the VF currently is not completely replaceable by structural information.

Recently, advanced iterations of the deep learning (DL) algorithm, which incorporates convolutional neural networks (CNNs) for visual recognition, have been widely adopted for glaucoma diagnosis^12–16^. A number of studies have demonstrated excellent glaucoma diagnostic ability using various clinical modalities. Some of those studies have reported excellent area under curve (AUC) of receiver operating characteristic (ROC)-based diagnostic performance of DL systems with fundus photography^16,17^. Asaoka et al. presented a DL model for discrimination of preperimetric glaucomatous visual field defect (VFD) from normal VF^18^. Even future VF test results were successfully predicted pointwisely by a DL model using only baseline VF test data^19^. Moreover, a recent studies showed very promising results for the prediction the severity of glaucomatous VFD from SD-OCT images^20^.

Thus, we developed a DL model for quantification of the MD of SAP from ODPs. The purpose of the present study was to validate the model’s diagnostic performance.

## Results

The total dataset included 1200 image pairs (ODPs and SAP results) for 563 eyes of 327 participants (254 eyes of 155 glaucoma patients, 309 eyes of 172 healthy controls). The subjects’ demographic characteristics are summarized in Table 1. The mean ages were 57.8 ± 13.7 years in the training set and 55.6 ± 9.1 years in the test set; 163 were women (57.8%) in the training set and 16 were women (35.6%) in the test set. Sex did not significantly differ between the two groups in either set (*P* = 0.982 and 0.115, respectively). Supplementary Table S1 describes the clinical characteristics according to glaucoma severity, based on MD (− 6 dB). The ICC for differential diagnosis showed excellent agreement (0.89, 95% CI [0.83, 0.94], *P* < 0.001).

**Table 1:** Descriptive statistics of study population.

	Training/validation set			Testing set		
	Normal	Glaucoma	*P*	Normal	Glaucoma	*P*
No. of Eyes (patients)	275 (155)	214 (127)	N/A	34 (17)	40 (28)	N/A
No. of images	483	517	N/A	81	119	N/A
Age (years)	55.8 ± 13.0	59.6 ± 14.0	< 0.001	55.5 ± 9.1	55.7 ± 8.9	0.937
Female (%)	89 (57.4%)	74 (58.3%)	0.982	9 (52.94%)	7 (25.00%)	0.115
IOP (mmHg)	13.8 ± 2.8	15.0 ± 3.3	< 0.001	14.2 ± 3.8	15.1 ± 2.9	0.569
SE (D)	− 2.4 ± 3.4	− 2.9 ± 3.5	0.078	− 1.3 ± 2.7	− 2.8 ± 2.9	< 0.001
CCT (μm)	546.0 ± 34.6	534.2 ± 33.3	< 0.001	542.8 ± 42.4	512.4 ± 31.7	0.004
SAP MD (dB)	0.1 ± 1.4	− 4.7 ± 4.5	< 0.001	0.3 ± 1.7	− 2.5 ± 3.5	0.001
SAP PSD (dB)	1.7 ± 0.5	6.9 ± 4.3	< 0.001	1.6 ± 0.2	4.9 ± 3.8	0.305

dB, Decibels; D, Diopters; CCT, central corneal thickness; SAP, standard automated perimetry; MD, mean deviation; PSD, pattern standard deviation.

**Figure 1:**
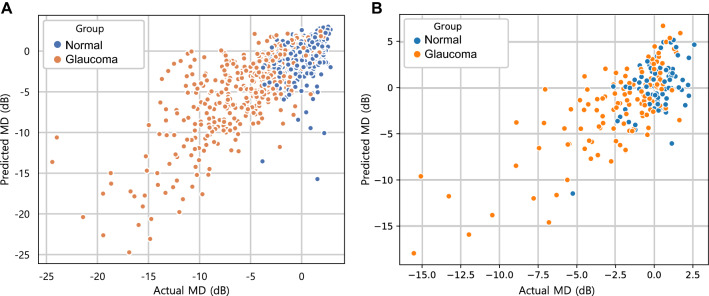
Scatterplot showing relationship between predicted mean deviation (MD) by deep learning (DL) model and actual MD observed from standard automated perimetry (SAP) in training/validation and testing datasets. (**A**) A strong correlation was found between the predicted and the observed MD in training/validation dataset (Pearson’s correlation coefficient = 0.748; R^2^ = 56.0%; *P* < 0.001). (**B**) Strong correlation also was observed in testing dataset (Pearson’s correlation coefficient = 0.755; R^2^ = 57.0%; *P* < 0.001).

**Figure 2:**
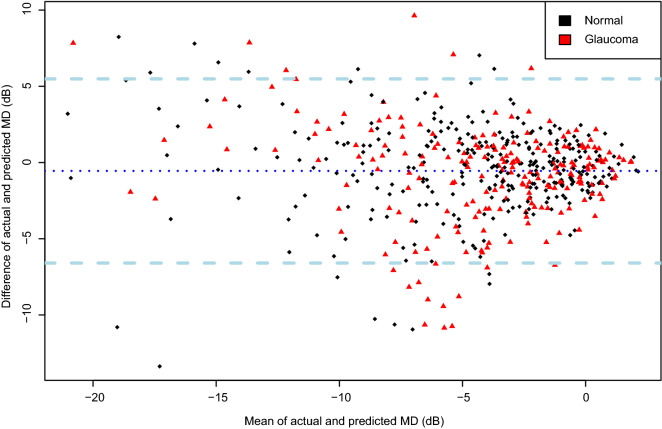
Bland–Altman plot demonstrating agreement between prediction and measurement of training/validation dataset. The predicted mean deviation (MD) showed good agreement with the actual measurement (95% confidence limits (CI) [− 5.25 dB, 5.15 dB]). No significant systemic bias was observed (bias = 0.1 dB, 95% CI [−  0.26, 0.06]).

The actual (SAP-based) MDs of the training and testing set were − 2.40 ± 4.13 dB and − 1.37 ± 3.96 dB, and the predicted (DL-based) MDs were − 2.50 ± 4.23 dB (*P* = 0.998; LMM) and − 1.27 ± 4.13 dB (*P* = 0.977; LMM), respectively. Also, strong agreement was observed in both datasets (MAE = 1.73 dB in the training set, 1.94 dB in the test set). A scatterplot demonstrated a strong correlation between the predicted and actual MDs (Training set: Pearson’s correlation coefficient = 0.748; R^2^ = 56.0%, Fig. 1A; Test set: Pearson’s correlation coefficient = 0.755; R^2^ = 57.0%, Fig. 1B). Figure 2 (training/validation set) and Supplementary Figure S1 (test set) provide a Bland–Altman plot evaluation of the agreement between the predictions and measurements. No evidence of systemic bias was observed in either dataset (bias = 0.09 [− 0.25, 0.07] dB (training set), bias = − 0.09 dB, 95% CI [− 0.44, 0.26] (test set)). In the subgroup analysis of the test set, the predicted MDs were 0.9 ± 1.98 and − 3.10 ± 4.02 dB in the normal and glaucoma groups, respectively, while the actual MDs were 0.28 ± 1.71 and − 2.51 ± 3.51 dB, respectively. To investigate the influence of the size of the training set on the DL performance, the same DL model was trained using only half of previous training set and tested. Stratified sampling was used to preserve the proportion of early to moderate-to-severe glaucoma in the total training set (3:1). After the training process, the correlation coefficient was 0.696 and the R^2^ score was 0.484 in the test set. The MAE was calculated to 2.47 dB.

In the additional fivefold cross-validation including the entire dataset (both the training and test sets), the mean R^2^ score and the MAE were 58.4 ± 2.06% and 1.95 ± 0.079 dB, respectively (Supplementary Table S2). The R^2^ score ranged from 54.9 to 60.8% and the MAE ranged from 1.87 to 2.10 dB.

**Figure 3:**
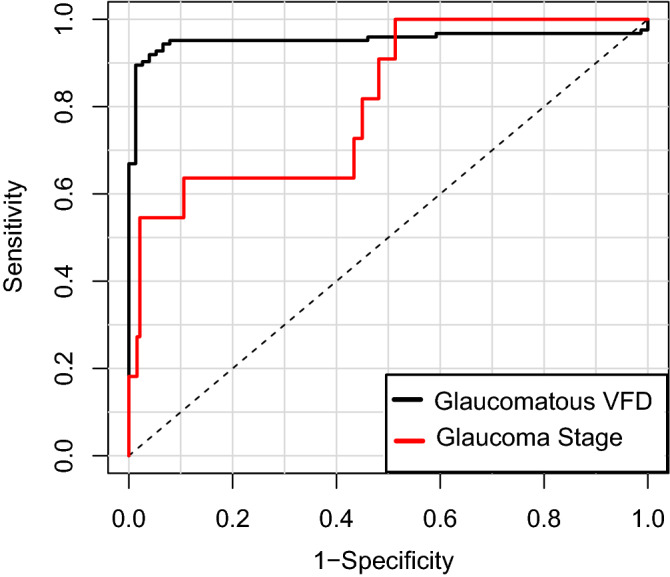
Receiver operating characteristic curve showing performance for detection of glaucomatous visual field defect (VFD) and discrimination of glaucoma stage according to mean deviation (MD; cutoff value: − 6 dB) in testing set. The areas under curve (AUCs) of the DL model were 0.953 (95% CI [0.919, 1.000]) for glaucomatous VFD detection and 0.812 (95% CI [0.716–0.999]) for glaucoma stage determination.

**Figure 4:**
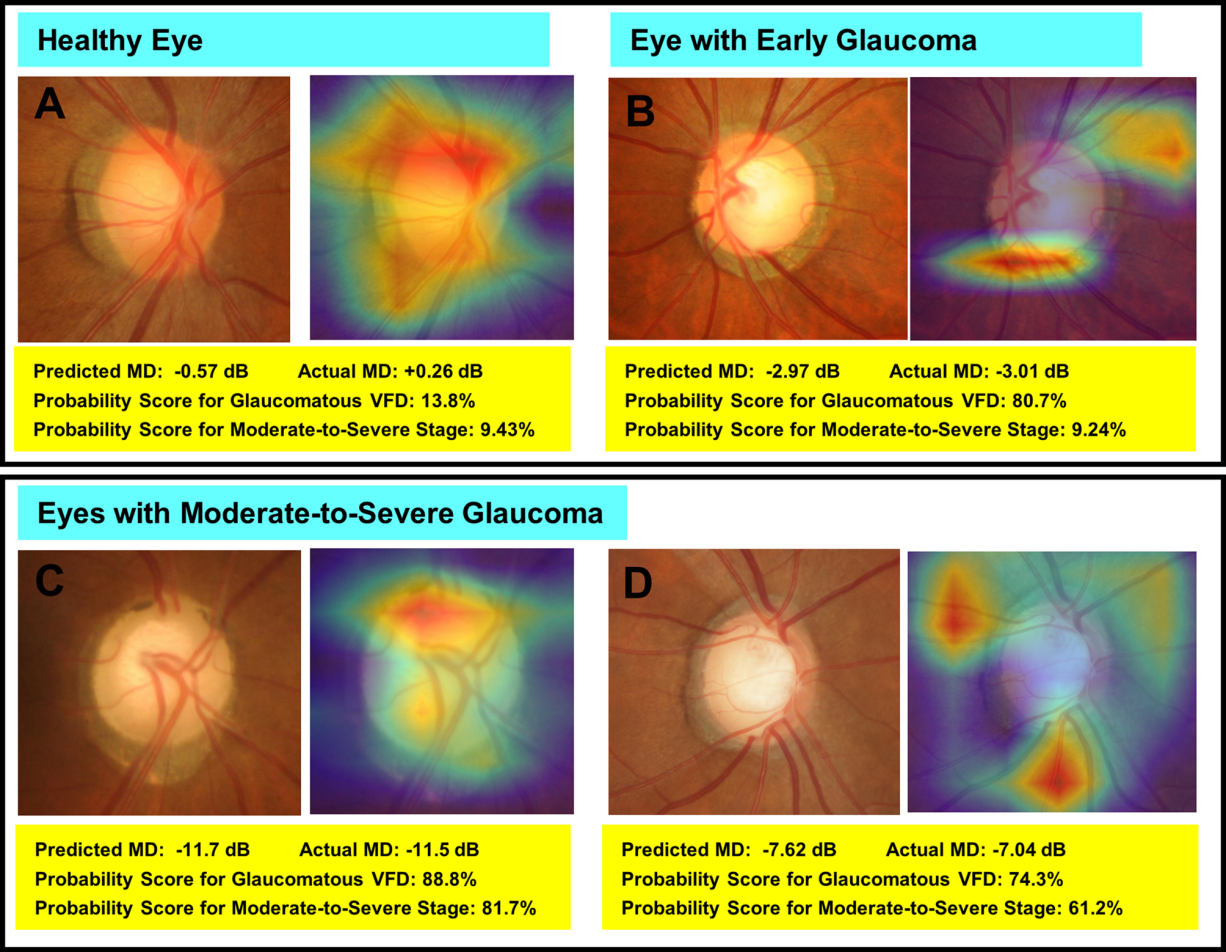
Class activation maps (heat maps) showing highly activated areas on optic disc photographs (ODPs) of healthy eye (**A**), early (**B**) and moderate-to-severe (**C**, **D**) stages of glaucoma based on which DL algorithm made its predictions. (**A**) A case of healthy eye. There is no visible neuroretinal thinning and no signs of glaucomatous VFD. The DL model excellently quantified the MD value. (**B**) In this early-glaucomatous eye, both the superior and inferior rims are thinned, and retinal nerve fiber layer (RNFL) loss is evident in the inferotemporal area. The MD value and glaucoma stage were accurately predicted. (**C**, **D**) Eyes of moderate-to-severe glaucoma. Neuroretinal rim thinning (more severe in (**D**)) with adjacent RNFL defect is shown. In both cases, the MD along with the presence of glaucomatous VFD and the glaucoma stage was correctly predicted. MD, mean deviation; VFD, visual field defect.

The ability of the DL model to differentiate eyes with glaucomatous VF loss from healthy eyes was excellent in both the training set (AUC = 0.986, 95% CI [0.980, 0.993]; Sensitivity 0.944 [0.925, 0.963], Specificity 0.975 [0.961, 0.988]) and the test set (AUC 0.953, 95% CI [0.919, 1.000]; Sensitivity 0.927 [0.879, 0.968]; Specificity 0.947 [0.895, 0.987]). Also, its ability to discriminate moderate-to-severe glaucoma was good (Training set: AUC 0.878, 95% CI [0.842, 0.913]; Sensitivity 0.711 [0.639, 0.777]; Specificity 0.929 [0.900, 0.954], Test set: AUC 0.860, 95% CI [0.771, 0.965]; Sensitivity 0.722 [0.590, 0.803]; Specificity 0.821 [0.714, 0.916]). In the subgroup analysis of the glaucoma group for discrimination of early versus moderate-to-severe glaucoma (excluding normal controls), the AUC was 0.847 [95% CI 0.813–0.881] in the training dataset, and 0.812 [95% CI 0.716–0.999] in the testing dataset. Figure 3 plots ROC curves showing the discriminating ability for glaucomatous VF loss from normal control (black curve), and early glaucoma from moderate-to-severe glaucoma (red curve) in the testing set. The ODPs with class activation maps of representative cases are provided in Fig. 4. Warmer colors (red > yellow > blue) represent a higher importance of the pixel to the classification task. The activated area was overlapped with clinically important areas including the neuroretinal rim and adjacent RNFL.

**Figure 5:**
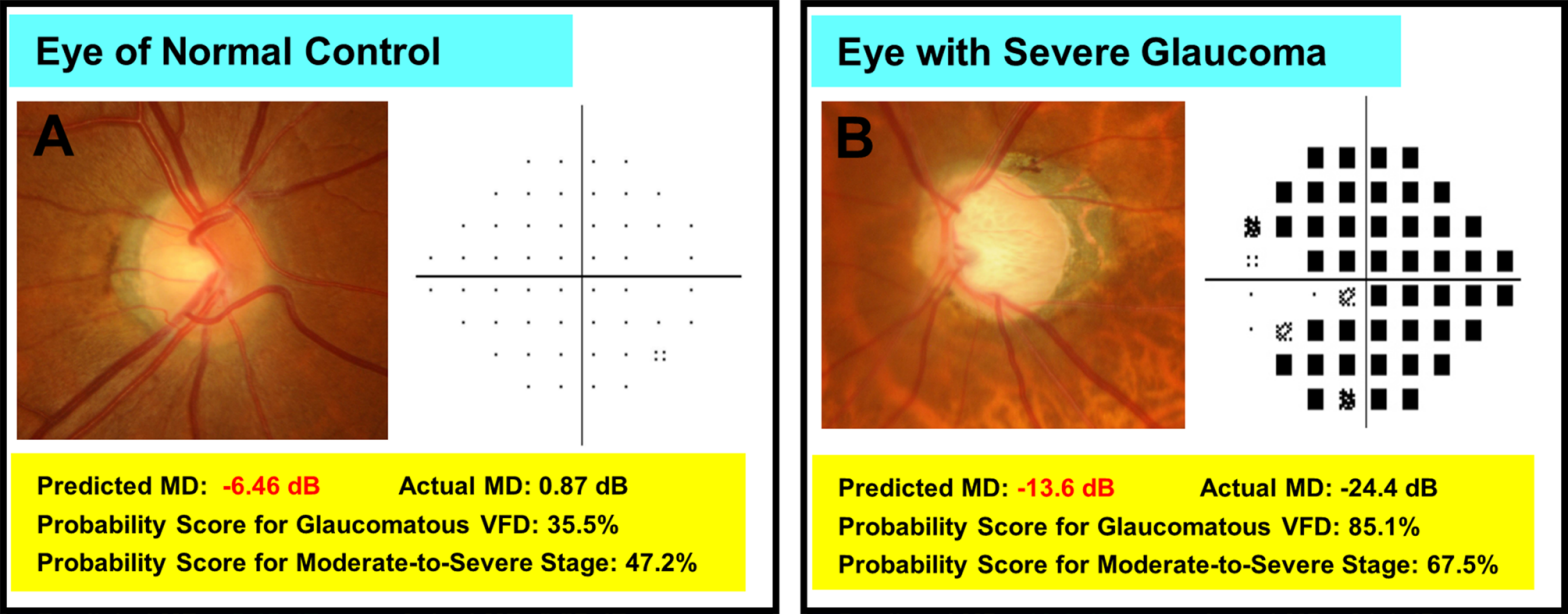
Random samples of optic disc photographs (ODPs) from which mean deviation (MD) was incorrectly predicted by deep learning algorithm. The ODPs are drawn with a pattern deviation plot (**A**) or a total deviation plot (**B**). (**A**) Example of healthy eye. (**B**) Case of severe glaucoma. The pattern deviation plot was not provided for the severely depressed field.

To identify the characteristics of the incorrect cases, the entire dataset was divided into 2 subgroups according to the ‘prediction error’ (defined as the absolute difference between the predicted and the actual MD), the cutoff value having been set to 5.30 dB in the training set and 4.60 dB in the test set according to the upper and lower limits of agreement on the Bland–Altman plot. The mean absolute differences between the predicted and the actual MD were 1.4 ± 1.2 dB (training set) and 1.7 ± 1.4 dB (test set) in the small prediction error group, and 7.9 ± 2.3 dB (training set) and 5.6 ± 2.2 dB (test set) in the large prediction error group. The results of the comparison are provided in Table 2. In the training set, the large prediction error group had higher IOP, lower MD, and higher PSD (*P* = 0.036, < 0.001, and < 0.001, respectively). In this group, accordingly, moderate-to-severe glaucoma was much more frequent (*P* < 0.001). In the test set, no clinical factors were found to be significantly different between the two subgroups. Some of the cases of incorrect prediction of MD by the DL model are shown in Fig. 5.

**Table 2:** Comparison between groups with small and large prediction errors, respectively, for mean deviation.

	Training/validation set			Testing set		
	Small prediction error*	Large prediction error	*P* value	Small prediction error*	Large prediction error	*P* value
No. of images	944	56	N/A	190	10	N/A
Age (years)	57.8 ± 13.6	57.4 ± 14.1	0.823	54.5 ± 8.7	59.0 ± 9.3	0.097
Sex (Female, %)	144 (58.3%)	19 (52.8%)	0.656	14 (41.2%)	2 (18.2%)	0.279
IOP (mmHg)	14.3 ± 3.0	15.8 ± 4.3	0.036	14.0 ± 3.3	14.9 ± 2.6	0.218
SE (D)	− 2.6 ± 3.5	− 3.5 ± 3.4	0.130	− 2.8 ± 3.0	− 1.5 ± 3.1	0.067
CCT (μm)	540.1 ± 34.4	535.5 ± 33.9	0.454	534 ± 36.1	529 ± 39.9	0.788
SAP MD (dB)	− 2.1 ± 3.7	− 8.0 ± 6.3	< 0.001	− 1.3 ± 2.9	− 2.1 ± 2.6	0.404
SAP PSD (dB)	4.1 ± 3.8	9.8 ± 5.5	< 0.001	3.7 ± 2.8	4.0 ± 3.1	0.691
Moderate-to-severe stage^†^	127 (13.5%)	39 (69.6%)	< 0.001	18 (9.5%)	2 (18.1%)	0.315

dB, Decibels; D, Diopters; CCT, central corneal thickness; SAP, standard automated perimetry; MD, mean deviation; PSD, pattern standard deviation.

*The group of which prediction error is equal to or smaller than 95% limits of agreement.

^†^MD lower than − 6 dB.

## Discussion

Our DL model for prediction of mean deviation from ODPs was validated in this study. The predicted and measured MDs showed strong correlation and good agreement. The class activation map highlighted the important region of interest of the DL model’s predictions.

There are several studies that have demonstrated the potential of DL models for glaucoma diagnosis using fundus photographs^13,16,17^, SD-OCT^21–23^, or VF tests^18^. However, most of the previously reported algorithms have focused only on detection of glaucomatous damage, in a “yes or no” fashion^16,17,24^. This approach required a time-consuming labeling process by human graders for ground truth, and was difficult to apply for detection of glaucoma progression. However, recent research has shown the possibility of overcoming the limitations of the simple classification problem using DL-based quantitative prediction of SD-OCT data such as RNFL thickness or BMO-MRW from ODPs^25,26^. However, in those studies, the input data (ODPs) and the outputs (SD-OCT data) were both structural information; indeed, in glaucoma diagnosis, it is imperative to consider comprehensive measurements including both structural and functional data. Thus prompted, we developed a DL algorithm to convert qualitative structural information (ODP) to quantitative functional data (SAP MD). Our DL model predicted the MD from ODPs with considerable accuracy in spite of the small study population.

Although the VF test is an essential modality for glaucoma diagnosis, it has been shown to be very vulnerable to test–retest variability. VF sensitivity can show short-term or long-term fluctuation^27^. The reliability of test results, therefore, is affected not only by the patient's ability to remain still during measurement, but also by measurement noise, even with high reliability indices^28^. This fact can diminish the ability to detect early-glaucomatous change and glaucoma progression. Considering VF variability, approximately 10 VFs are required to obtain reliable point-wise VF sensitivity and mean sensitivity^29^. On the other hand, ODPs and red-free RNFL photographs are easy to take, and offer relatively reliable results via appropriate pupil dilation. Even when photographs are taken mistakenly, the problem is easily noticed by technicians, who subsequently can easily retake the photographs. Also, fundus photography is still the main modality for many glaucoma-screening centers. According to the results of the present study, the DL algorithm showed the potential to be a useful tool for estimation of the global index of SAP in screening centers where SAPs are unavailable. Furthermore, as the model provides quantitative values, it can be applied to detection of glaucoma progression by trend-based analysis using estimated MDs with serial ODPs. Further training with larger datasets including longitudinal observations of each participant would help to elucidate this conjecture.

To estimate the generalizability of the method with the limited number of samples, we investigated the range distribution of the performance values by fivefold CV. Considering that the standard deviation was relatively small and that the worst-case values (R^2^ score of 54.9% and MAE of 2.10 dB) were comparable to the mean values, we concluded that the prediction performance of the DL model might be robust to data heterogeneity. Based on this, we determined the feasibility of the robustness of this model with larger observations and concluded that the generalization potential of this method might be good. However, confirmation of the generalizability of this model will have to await further study.

Typically, the DL performance for a test set becomes worse than that for a training dataset. However, the results in this study showed a slightly better correlation between the predicted and observed MDs in the test subset relative to the training set. This result might mean that the diagnostic performance and loss function (mean square error) had not converged completely before the end of the training. However, when we thoroughly reviewed the learning curve of the DL model, the loss function converged and stopped decreasing at the end of the training. Hence, it might be a coincidence that test performance was higher than the training performance. Another possible explanation is the dropout technique adopted in the training. In the training process, some of neurons were disabled, so that the training loss (or prediction error) could have been higher due to the task for the artificially weakened network having become harder. In contrast, during the testing process, all of the neurons were available, and so the network was fully capable and might have perform better than in training. Finally, the discrepancy may be caused from the distribution of early and severe glaucoma in two datasets. In subgroup analysis, we revealed lower SAP MD was associated to large prediction error. Since the SAP MD of testing set was greater than that of training set, it could cause such a difference between train/test results. Indeed, in the subsequent fivefold CV with entire dataset, the R^2^ score became better (58.4%) than that from fivefold CV with only training dataset (56.0%). We cannot identify the exact reason of the discrepancy of correlation between training and testing sets, however, the three hypotheses may explain it in part.

Taking all of this one step further, we modified our quantitative prediction model for three classifiers. The first classifier judges the given ODP as having glaucomatous VFD or not. This is a typical classification problem that many other studies have tackled; in the present study, moreover, the discriminating performance was shown to be excellent. The second classifier generates a heat map of the MD prediction model. Its generated class activation maps showed that the DL model had generally focused on the superior or inferior neuroretinal rim and adjacent RNFL, as in diagnosis by physicians and clinicians. The inner part of the optic disc cup and the far-peripheral area near the image boundary were hardly activated. This suggests that the DL model interpreted the image based on clinically important rather than unaccountable information, which would give clinicians more confidence in accepting the output of the DL model. And in fact, in the current study, the ability to discriminate moderate-to-severe glaucoma from normal or early glaucoma was judged to be good. The third DL classifier discriminated moderate-to-severe glaucoma from early glaucoma. It also showed good diagnostic accuracy in the training and test sets in terms of AUC.

In this study, we formulated the model only for prediction of MD, not also PSD. PSD values are calculated on the basis of the variation from the normal hill of vision involving the total deviation plot. Although PSD has its own diagnostic strength for glaucoma, in advanced-stage glaucoma with the overall reduction in sensitivity, the PSD value decreases. This paradoxical decrease complicates the introduction to glaucoma staging^30^. Therefore, PSD was not employed in the training or prediction process of the proposed model.

In the subgroup analysis, we observed that higher IOP, lower MD, higher PSD, and more severe stage (MD < − 6 dB) were associated with large prediction error in the training/validation set. Because the number of subjects in the large prediction error group in the test set was too small for statistical comparison, we could analyze the factors associated with large prediction error only in the training/validation set. Considering that most of the subjects of the glaucoma group in this study were in the early stage, we deduced that the numbers for the moderate (147 pairs) and severe (42 pairs) stages were not sufficient to adequately train the DL model. To upbuild this model and make it effective for detection of glaucoma progression in the severe stage, it would be necessary to enroll more moderate-to-severe glaucoma patients.

Our study has several limitations. First, the dataset was relatively small for training of a DL network, which forced us to adopt transfer learning only. The prediction error of a DL model can be decreased by training of a DL network on a larger dataset. Indeed, we revealed that the testing performance can be dependent on the size of the training set. The prediction error of the DL model trained with half of the entire training set was larger than that of the original DL model, and when the testing set with the better MD was incorporated into the training/validation set, the validation performance improved. Especially, more participants with moderate-to-severe glaucoma will drastically improve the prediction error for a large prediction error group. Second, most of the subjects were of Korean descent, and the majority of the glaucoma patients had normal-tension glaucoma rather than high-tension glaucoma, which fact reduces the generalizability of the present results. Third, we validated only the global MD prediction of the DL model, not other factors, such as visual field index (VFI) or point-wise sensitivity. Although the first modified classifier detected the glaucomatous VFD in the aspect of PSD and GHT, it is not a quantitative prediction model. To develop and validate the model for other VF indices trained with a larger population would be interesting. Fourth, media opacities, which can influence MD, were not taken into account in this study. Although we had introduced the inclusion criterion of BCVA ≥ 20/40 to exclude eyes with severe media opacities, the exact status regarding the media opacities of the enrolled eyes was unknown. Fifth, because we used ODPs for training and testing, the information included in the fovea and RNFL apart from the optic disc area was not considered. This loss of information might have affected the diagnostic accuracy. Comparison of DL performance between entire fundus photography and optic disc photography would be an interesting topic for future investigation. Sixth, although only reliable VF results were included, MD variability nonetheless can make the DL model’s training process more difficult. Indeed, the MD test–retest variability in the normal group was as high as 3.2 dB. Taking this into account, the MAE of this model (1.73 dB in the training/validation set, 1.94 dB in the test set) might be more understandable. Seventh and finally, given that we diagnosed glaucoma by both structural and functional defects, we did not consider preperimetric glaucoma.

In conclusion, the trained DL algorithm showed excellent performance for discrimination of glaucomatous VFD and successfully quantified SAP MD, with a strong measurement correlation and good agreement. Moreover, it showed a potential for complementation of VF assessment and detection of glaucoma progression. With further training in a larger dataset, it could provide useful information for VF loss in cases where VF test results are unreliable due to a lack of patient concentration or other reasons.

## Methods

The present research followed the tenets of the Declaration of Helsinki, the study protocol having been approved by the local ethical committee of Seoul National University Hospital. Informed consent was waived by the Seoul National University Hospital (SNUH) Institutional Review Board due to the retrospective nature of the study.

### Subjects

The subjects for the training/validation set were chosen from Seoul National University Hospital (SNUH)’s Glaucoma Clinic database representative of the years 2009 to 2018. To evaluate the robustness of the DL model, an external test set was recruited from the *Gangnam Eye Study*, an ongoing cohort study conducted at the Gangnam Healthcare Center (GHC) at Seoul National University Hospital. All of the subjects underwent common ophthalmologic examinations, including best-corrected visual acuity measurement, refraction, intraocular pressure (IOP) measurement by Goldmann applanation tonometry, corneal pachymetry (Pocket II Pachymeter Echo Graph; Quantel Medical, Clermont-Ferrand, France), slit-lamp biomicroscopy, gonioscopy, and dilated stereoscopic optic disc examination^31^. They additionally underwent stereo optic disc photography, red-free RNFL photography (SNUH: Vx-10a; Kowa Optimed Inc., Tokyo, Japan or Visucam 524; Carl Zeiss Meditec, Dublic, CA, USA; *Gangnam Eye Study*: EOD D060; Canon Inc., Utsunomiya, Japan), as well as SAP using the Swedish interactive threshold algorithm according to the central 30–2 or 24–2 standard program (Humphrey Field Analyzer II; Carl Zeiss Meditec, Dublin, CA, USA)^32^.

The inclusion criteria were as follows: age between 20 and 80, best corrected visual acuity of ≥ 20/40, no history or evidence of retinal diseases (diabetic retinopathy, macular degeneration, retinal detachment, epiretinal membrane) or nonglaucomatous optic nerve diseases, and no history of treatment that might cause retinal or optic nerve damage (i.e., chloroquine, ethambutol), laser therapy, or ocular surgery except uncomplicated cataract surgery. Individuals with high myopia (AL ≥ 27.50 mm) or severe media opacity that would significantly obscure ODPs or affect the VF test results were excluded.

### Diagnosis of glaucoma

Diagnosis of glaucoma was based on the presence of glaucomatous VF loss on SAP, which was defined as a pattern standard deviation (PSD) < 5% or glaucoma hemifield test (GHT) results outside the normal limits, and on the presence of glaucomatous optic disc cupping (i.e. neuroretinal rim thinning, notching, excavation) or RNFL defect. VF defects had to be repeatable on at least 2 consecutive tests. Visual fields were excluded if they had more than 20% fixation losses or more than 15% false-positive or false-negative errors. The control subjects had an IOP ≤ 21 mmHg with no history of increased IOP, no glaucomatous disc appearance or RNFL defect, and a normal VF on SAP. The glaucomatous change of the optic disc and that of the RNFL were assessed by three certified glaucoma specialists each with over 8 years of experience (Y.K.K., J.W.J., and K.H.P.) who were masked to all other information on the eyes. The enrolled eyes were diagnosed as glaucoma only if the patterns of visual field defect correlated with glaucomatous structural defect. If the opinions on the diagnosis of glaucoma differed, the pertinent eyes were excluded from further analysis.

### Design of deep learning model

A DL algorithm was trained to predict SAP MD from an assessment of ODPs. Since the target value was the MD from SAP, the ODP and SAP MD from the same eye were inputted as a train-target pair. The pairs of photographs and OCT scans acquired from SNUH were assigned as the training/validation set (1000 pairs), and those acquired from GHC were assigned as the testing set (200 pairs).

Image preprocessing was performed to make the ODPs suitable for the DL algorithm. The resolution of the images was converted to 331 × 331 pixels (1600 × 1216 pixels to 2214 × 1660 pixels originally) and the pixel values were scaled to within the range of 0–1. Data augmentation (horizontal flip, horizontal shifting [< 10% of image size]) and image scaling (95–100% of original size) were performed to make the DL algorithm more robust to heterogeneity.

Considering that this was a small-scale study for deep learning, we adopted a pre-trained deep neural network for feature extraction and transfer learning. Since the study population was relatively small for training of the entire DL network, the weights of the pretrained CNNs were frozen so as not to be trained further by our dataset. In particular, we adopted the NASNet (‘NAS’ is an abbreviation of neural architecture search) architecture to extract rich features from each ODP^33,34^. In brief, the network consisted of normal cells and reduction cells. Normal cells are convolutional cells returning a feature map of the same dimension, whereas reduction cells are convolutional cells that return a feature map the height and width of which are reduced by a factor of two. The detailed architecture of the convolutional cells has been determined by the reinforcement learning search method^34^. This CNN has been shown to have superior discriminating ability for computer vision tasks compared with other CNNs such as Inception-v3. Since NASNet was originally designed for discrimination of ordinary objects rather than ODPs, we used only the convolution layers for feature extraction and discarded the final classification layer.

For the purposes of a regression model, the feature vector extracted from the CNN was inputted to the subsequent hidden layers including 30 and 15 neurons each and a final layer with single neuron. The mean square error was used as the loss function, and the Nesterov-Adam optimizer (learning rate: 0.003) was introduced for training of the fully connected network. To prevent overfitting, dropout (rate: 0.6) and L2 regularization (λ = 0.015) were applied in the training process.

### Deep learning classifier for discrimination of glaucomatous visual field loss

For visualization of important areas for DL prediction, class activation maps (heat maps) have been widely adopted^35^. However, heat maps are currently available only in the classification problem, not the regression problem. Hence, three additional DL classifiers were constructed. First, we formulated a classifier to discriminate eyes with glaucomatous VF loss from healthy eyes. Second, we constructed a DL classifier to discriminate eyes with moderate-to-severe glaucoma (MD <  − 6 dB) from eyes of the normal group or with early glaucoma (MD ≥  − 6 dB), and validated its diagnostic accuracy. Third, a DL classifier similar to the second one but to discriminate moderate-to-severe glaucoma from early glaucoma was built. For the training and testing of this last classifier, only glaucomatous eyes were used (not normal controls). Each classifier used the same feature vectors of the given ODPs extracted from the CNN, which were also inputted to the DL regression model. All of the classifiers had only one hidden layer with [5, 10, 15] neurons, along with the final softmax classifier to output the probability for glaucomatous VFD (in the first model) or moderate-to-severe stage (in the second and third models). The number of neurons in the hidden layer was determined during the tuning process with cross-validation, using only the training/validation dataset. The Nesterov-Adam optimizer (learning rate: 0.003) also was introduced for training of the DL classifiers along with dropout and L2 regularization to prevent overfitting. The heat map was generated from the second DL classifier, which is closer to the MD prediction model than is the first classifier.

### Statistical analysis

Among the control and glaucoma groups, demographic and SAP parameters were compared by the linear mixed model (LMM) accounting for multiple measurements per patient. A 2-way random effects model was applied to determine the inter-observer reproducibility of the estimate of agreement among the 3 raters, and the intra-class coefficient (ICC) was calculated. We evaluated the performance of the DL algorithm for prediction of MD by correlation coefficient and mean absolute error (MAE) between the predictions and the actual MDs. Five-fold cross-validation (CV) was performed for validation and hyperparameter tuning. To that end, the dataset was divided into five equally sized subsets, and four of the five arms were used to train the machine learning model without memorizing the remaining arm; the remaining arm was then used to validate the diagnostic performance^36^. This process was repeated five times to assure that each arm was used as a validation set once. After the training process completed, the test set was used to confirm the DL model’s predictions.

To obtain a more detailed understanding of the potential generalization performance of this DL model, we performed another fivefold CV with the entire dataset (training/validation set and testing set) to reveal the range of the R^2^ score and MAE. We speculated that the differences between the testing performances in each CV would be useful in estimating the robustness of this method.

The discriminating ability for glaucomatous VF loss was determined by the AUC of the ROC analysis with 95% confidence intervals (CI). The sensitivity and specificity values were computed at the optimal cut-off value that maximized the Youden index (obtained as *J* = [sensitivity + specificity − 1])^37^.

All of the statistical analyses were performed using R software (version 3.5.2) for statistics. P values less than 0.05 were considered statistically significant. The data ranges were recorded as mean ± standard deviations.

## Supplementary information

Supplementary information.

## Data Availability

The dataset generated during the current study is available from the corresponding author on reasonable request.
