# Supplementary material for: Estimating visual field loss from monoscopic optic disc photography using deep learning model
Source: Sci Rep. 2020 Dec 3;10:21052. doi: 10.1038/s41598-020-78144-1 (PMC7712913; doi:10.1038/s41598-020-78144-1)
Supplement: Supplementary file 1 — Supplementary information. [file 41598_2020_78144_MOESM1_ESM.pdf]

Estimating Visual Field Loss from Monoscopic Optic Disc Photography using Deep Learning Model

Jinho Lee, Yong Woo Kim, Ahnul Ha, Young Kook Kim, Ki Ho Park, Hyuk Jin Choi, Jin Wook Jeoung

Supplementary Table S1. Descriptive Statistics of the According to Glaucoma Severity in the Training and Testing Set

	Training/Validation Set			Testing Set		
	Early Stage*	Moderate-to-Severe Stage	P	Early Stage*	Moderate-to-Severe Stage	P
No. of Eyes (patients)	162 (101)	52 (26)	N/A	31 (23)	9 (5)	N/A
No. of images	372	145	N/A	99	20	N/A
Age (years)	55.8 ± 13.0	59.6 ± 14.0	0.041	54.2 ± 8.6	59.1 ± 5.7	0.008
Female (%)	54 (53.5%)	20 (76.9%)	0.167	5 (21.7%)	2 (40.0%)	0.574
IOP (mmHg)	14.1 ± 3.5	15.1 ± 3.1	0.008	14.2 ± 3.2	15.2 ± 2.8	0.132
SE (D)	-2.7 ± 3.0	-3.0 ± 2.8	0.175	-2.5 ± 3.0	-3.2 ± 3.1	0.062
CCT (μm)	535.2 ± 33.5	525.5 ± 40.2	<0.001	522.1 ± 33.2	501.4 ± 35.6	<0.001
SAP MD (dB)	-2.2 ± 2.0	-10.1 ± 3.5	<0.001	-1.6 ± 1.9	-10.0 ± 3.1	<0.001
SAP PSD (dB)	4.8 ± 2.8	11.6 ± 3.3	<0.001	4.7 ± 2.5	10.4 ± 2.1	<0.001

* Mean deviation ≥ -6 dB.

dB = Decibels; D = Diopters; CCT = central corneal thickness; SAP = standard automated perimetry; MD = mean deviation; PSD = pattern standard deviation.

Supplementary Table S2. Performance metrics of deep learning algorithm for quantification of mean deviation (MD) of standard automated perimetry (SAP) by 5-fold cross-validation of entire dataset

	CV #1	CV #2	CV #3	CV #4	CV #5	Mean	SD
R^2 score (%)	59.1%	54.9%	59.9%	60.8%	57.7%	58.4%	2.06%
MAE (dB)	1.96	2.10	1.91	1.87	1.92	1.95	0.079

CV = cross-validation; SD = standard deviation; MAE = mean absolute error.

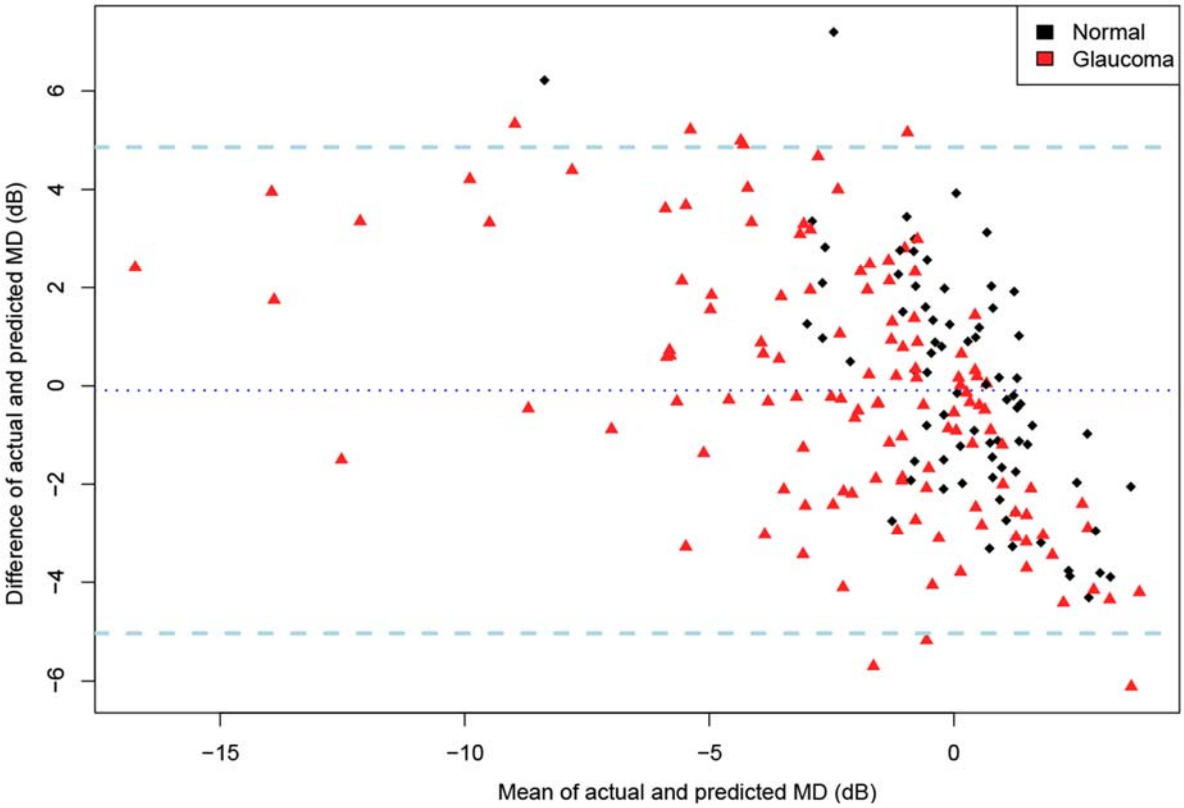

Supplementary Figure S1. Bland-Altman plot demonstrating agreement between prediction and measurement of the test dataset. The predicted mean deviation (MD) showed good agreement with the actual measurement (95% confidence limits (CI) [-4.26 dB, 4.86 dB]). No significant systemic bias was observed (bias = -0.09 dB, 95% CI [-0.44, 0.26]).
